# Serum cystatin C and mild cognitive impairment: The mediating role of glucose homeostasis

**DOI:** 10.3389/fnagi.2023.1102762

**Published:** 2023-03-28

**Authors:** Kai Li, Jing Xu, Meiduo Zhao, Jingtao Wu, Yayuan Mei, Quan Zhou, Jiaxin Zhao, Yanbing Li, Ming Yang, Qun Xu

**Affiliations:** ^1^Department of Epidemiology and Biostatistics, Institute of Basic Medical Sciences Chinese Academy of Medical Sciences, School of Basic Medicine Peking Union Medical College, Beijing, China; ^2^Center of Environmental and Health Sciences, Chinese Academy of Medical Sciences, Peking Union Medical College, Beijing, China

**Keywords:** mild cognitive impairment, glucose homeostasis, cystatin C, diabetes, population-based study

## Abstract

**Background:**

This study explored the mediating role of glucose homeostasis indicators in the relationship between serum cystatin C and mild cognitive impairment (MCI).

**Methods:**

The present study used a cross-sectional design and included 514 participants aged ≥50 years in Beijing, China. The Mini-Mental State Examination was used to assess cognitive function. Serum cystatin C and a comprehensive set of glucose homeostasis indicators were detected, including fasting blood glucose (FBG), glycosylated albumin percentage (GAP), glycated hemoglobin (HbAlc), insulin, and homeostatic model assessment of insulin resistance (HOMA-IR), and beta cell function (HOMA-β). Generalized linear models were used to investigate the associations among cystatin C, glucose homeostasis indicators, and cognitive function. Mediation analysis was conducted to explore potential mediator variables.

**Results:**

In this study of 514 participants, 76 (14.8%) had MCI. Those with cystatin C levels ≥1.09 mg/L had a 1.98-fold higher risk of MCI than those with levels <1.09 mg/L (95% CI, 1.05–3.69). FBG, GAP, and HbA1c increased the risk of MCI, while HOMA-β decreased the risk. Notably, the associations between MCI risk and cystatin C or glucose homeostasis were only founded in diabetes patients. Serum cystatin C was found to be positively associated with HOMA-β (beta (95% CI): 0.20 [0.06, 0.34]), HOMA-IR (0.23 [0.09, 0.36]), and insulin (0.22 [0.09, 0.34]) levels. Moreover, HOMA-β was identified as playing a negative mediating role (proportion mediated: −16%) in the relationship between cystatin C and MCI.

**Conclusion:**

Elevated levels of cystatin C are associated with an increased risk of MCI. The glucose homeostasis indicator, HOMA-β, plays a negative mediating role in the relationship between cystatin C and MCI risk.

## 1. Introduction

Dementia is a major cause of disability in the elderly population worldwide, with an estimated 135 million cases projected by 2050 (GBD 2019 Dementia Forecasting Collaborators, 2022). Mild cognitive impairment (MCI) represents the prodromal stage of dementia, with a progression rate of 20 to 60% within 2–4 years (Ward et al., 2013). Therefore, MCI serves as a critical target for interventions aimed at preventing or delaying the onset of dementia.

Cystatin C is a cysteine protease inhibitor produced by all nucleated cells (Benndorf, 2018) and has a broad spectrum of biological roles (Bobek and Levine, 1992; Filler et al., 2005). However, the association between cystatin C and cognitive impairment remains unclear. On the one hand, cystatin C has been suggested as a neuroprotective factor (Gauthier et al., 2011), exerting its neuroprotective effect through mechanisms such as the inhibition of cysteine proteases (Bernstein et al., 1996), induction of neurogenesis (Sun, 1989), induction of autophagy (Tizon et al., 2010), inhibition of oligomerization (Olafsson et al., 1996), and amyloid fibril formation (Olafsson et al., 1996). A study in Neurology demonstrated that an increase in cystatin C activity could prevent the development of Alzheimer’s disease, and every 0.1 μmol/L decrease in cystatin C level increased the risk of Alzheimer’s disease by 29% (HR 1.29, 95% CI 1.03−1.63, *p* < 0.03) (Sundelof et al., 2008). On the other hand, some population-based studies have found a negative correlation between cystatin C levels and cognitive function. For example, in a study involving 6,869 individuals, Cui et al. (2020) found that higher levels of cystatin C were associated with cognitive impairment, with an OR (95% confidence interval, CI) of 1.56 (1.10, 2.22) (Cui et al., 2020). A study conducted in a Japanese community also observed a significant negative correlation between cystatin C levels and MMSE scores (Kono et al., 2017). The reason for these contradictory results is currently unclear. Previous studies have often focused only on the direct effect of cystatin C on cognitive function, while ignoring other potential factors that may have indirect effects. The glucose homeostasis proposed in this study is one such potential influencing factor.

Abnormal glucose homeostasis is widely recognized as a significant risk factor for cognitive impairment (Luchsinger et al., 2004; Bellia et al., 2022). Interestingly, previous research has also established an association between cystatin C and glucose homeostasis. A 15 years cohort study demonstrated that, after controlling for multiple covariates, baseline serum cystatin C was significantly associated with 15 years cumulative incidence of type 2 diabetes (OR per log of cystatin C unit 2.19, 95% CI 1.02–4.68) (Sahakyan et al., 2011). Additionally, a study conducted in Western New York demonstrated that individuals with high levels of cystatin C had a threefold increased risk of progressing to prediabetes (Donahue et al., 2007). These findings suggest that glucose homeostasis is not only associated with cystatin C but also with cognitive impairment, and therefore, may play a crucial role in the relationship between cystatin C and cognitive impairment.

The aim of this study is to investigate the potential relationship between various glucose homeostasis indicators, cystatin C, and cognitive impairment. Furthermore, we aim to explore whether glucose homeostasis indicators mediate the relationship between cystatin C and the risk of cognitive impairment through mediation analysis.

## 2. Materials and methods

### 2.1. Study design and population

The present study had a cross-sectional design and used data from the 2021 follow-up survey of a cohort study conducted in Beijing, China. Data from four communities in three administrative regions of Beijing (Miyun, Daxing, and Fangshang) were included in this analysis. The study was approved by the Institutional Review Board of the Institute of Basic Medical Sciences, Chinese Academy of Medical Sciences.

We recruited participants through neighborhood committee mobilization and telephone notification. To be eligible for the study, participants had to meet two criteria: they must be at least 50 years of age, and they must have lived in the local community for at least 5 years. To minimize bias resulting from confounding factors, we excluded individuals who were unable to complete the questionnaire because of medical reasons or those whose blood samples were not retained. All eligible participants signed an informed consent form before the questionnaire surveys, anthropometric measurements, cognitive function tests, and sample collection were conducted by professional staff.

A stable team of 15 professionals, including qualified physicians, trained investigators, and certified nurses, was established. The physicians conducted anthropometric measurements. The trained investigators conducted face-to-face questionnaire surveys (including demographic information, lifestyle, disease history, medication history, and cognitive function tests). The certified nurses collected blood samples. Prior to fieldwork, we trained all staff members to help them master the standard operating procedures.

### 2.2. Assessment of cystatin C and glucose homeostasis indicators

We use a comprehensive set of indicators to assess glucose homeostasis, including fasting blood glucose (FBG), glycosylated albumin percentage (GAP), glycated hemoglobin (HbAlc), insulin, homeostatic model assessment of insulin resistance (HOMA-IR), and homeostatic model assessment of beta cell function (HOMA-β) (Li et al., 2021).

The participants were contacted by telephone on the day before visit and were reminded to fast strictly to ensure that biochemical indicators such as blood glucose were not affected by diet. Fasting venous peripheral blood samples of all participants were collected during the same time period (8:00–9:00) to control for the circadian rhythms of the body. Procoagulation tubes (BD Vacutainer^®^ SST™ II, Becton Dickinson, USA) and anticoagulation tubes (BD Vacutainer^®^ K2 EDTA, Becton Dickinson, USA) were used to collect fasting serum and whole blood samples from the participants, respectively, and tubes with a thixotropic gel barrier (BD Vacutainer^®^ SST™ II, REF 367955) were used for serum biochemical analyses, including those for cystatin C (mg/L), glucose (mmol/L), glycated albumin (g/L), albumin (g/L), and insulin (mU/L). Fasting blood glucose, glycated albumin, and albumin were measured using a Beckman Coulter analyzer (AU5800 Analyzer, Beckman Coulter, Brea, CA, USA), and insulin level was measured using a Siemens ADIVA Centaur XP analyzer (Siemens Healthcare Diagnostics Inc., Tarrytown, NY, USA). HbA1c levels (%) were also measured at each examination through ion-exchange high-performance liquid chromatography (Bio-Rad D-100, Bio-Rad, Hercules, CA, USA). GAP was calculated by dividing the amount of glycated albumin by that of albumin. FBG concentration was analyzed using an enzymatic colorimetric method in conjunction with the hexokinase photometric method. Insulin concentrations were determined through a chemiluminescent immunoassay. Albumin and glycated albumin concentrations were determined using the bromocresol green method and bromocresol violet method, respectively. All laboratory operations were performed at the Department of Clinical Laboratory of the Peking Union Medical College Hospital. We obtained the homeostasis model assessment of insulin resistance (HOMA-IR) and homeostasis model assessment of islet beta cells function (HOMA-β) to quantitatively evaluate insulin resistance and islet beta cell function, respectively, which can be mathematically expressed as follows (Matthews et al., 1985):


H⁢O⁢M⁢A-I⁢R=

[f⁢a⁢s⁢t⁢i⁢n⁢g⁢g⁢l⁢u⁢c⁢o⁢s⁢e⁢(m⁢m⁢o⁢l/L)×i⁢n⁢s⁢u⁢l⁢i⁢n⁢(m⁢U/L)]/22.5

H⁢O⁢M⁢A-β=

i⁢n⁢s⁢u⁢l⁢i⁢n⁢(m⁢U/L)×20/[f⁢a⁢s⁢t⁢i⁢n⁢g⁢g⁢l⁢u⁢c⁢o⁢s⁢e⁢(m⁢m⁢o⁢l/L)-3.5]

Higher HOMA-IR values indicate higher levels of insulin resistance and lower HOMA-β values indicate poorer islet beta cell function.

### 2.3. Measurement of cognitive function

We used the Mini-Mental State Examination (MMSE) in this study to assess cognitive function (Folstein et al., 1975). The MMSE consists of 30 questions, each assigned one point, with a total of 30 points. Based on their MMSE scores, the participants were classified into two categories: normal cognitive function and MCI. In order to detect MCI, the present study considered the influence of education level on the MMSE test and determined precise cutoff values as follows: individuals with a junior high school education or above were categorized as having MCI if they scored ≤24 on the MMSE, those with a primary school education were classified as having MCI if their score was ≤20, and those who were illiterate were classified as having MCI if their score was ≤17 (Li et al., 2016).

### 2.4. Covariates

In this study, hypertension was defined as a mean of SBP ≥140 mm Hg, a mean of DBP ≥90 mm Hg, self-reported history of hypertension, or the use of antihypertensive agents. Diabetes status was verified by fasting blood glucose (FBG) ≥7.0 mmol/L, HbA1c ≥6.5%, a self-reported history of diabetes, or the use of antidiabetic drugs. Body mass index (BMI) was calculated by dividing a person’s weight in kilograms by height in meters squared. Obesity status was defined according to BMI; BMI <18.5 was considered underweight, 18.5≤ BMI <24 was considered normal, 24≤ BMI <28 was overweight, and BMI ≥28 was considered obese. Smoking status and drinking status were categorized as never, former, or current. Education levels were divided into three categories: illiterate, primary school, and junior high school and above. Annual household income was divided into three categories <10, 10–100, and >100 (unit: 1,000 Chinese yuan/year). Serum cystatin C ≥1.09 mg/L was defined as elevated cystatin C (Muntner et al., 2009).

### 2.5. Statistical analysis

#### 2.5.1. Descriptive analysis

Continuous variables are expressed as mean ± standard deviation (SD), and categorical variables are expressed as percentage (%) in this study. One-way analysis of variance (ANOVA), unpaired *t* tests, and chi-square tests were used to perform analyses. Spearman coefficients were calculated for the correlation between MMSE scores and glucose homeostasis indicators. Natural logarithm transformation was performed when necessary for the continuous variables that did not fit the normal distribution.

#### 2.5.2. Model construction

**FIGURE 1:**
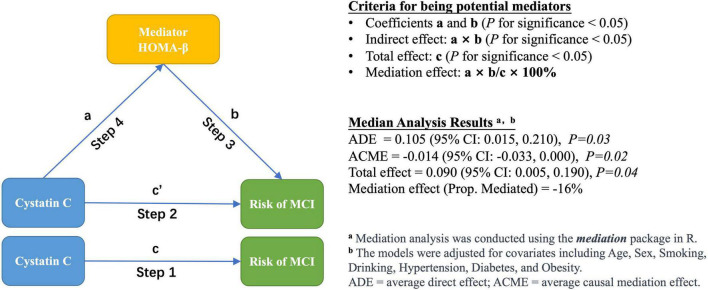
Modeling strategies and mediation analysis.

As shown in Figure 1, we employed a four-step modeling strategy to investigate the association between cystatin C, glucose homeostasis indicators, and the risk of MCI. In all models, cystatin C was categorized into two groups (≤1.09 and >1.09 mg/L) (Muntner et al., 2009).

Step 1: The association between cystatin C and the risk of MCI was assessed using logistic regression models. The following covariates were examined as potential confounders: age, sex, education, household income, smoking status, drinking status, hypertension status, and obesity status.

Step 2: Based on Step 1, various glucose homeostasis indicators were additionally adjusted in the model to investigate the indirect effect of cystatin C on the risk of MCI.

Step 3: The association between glucose homeostasis indicators and the risk of MCI was evaluated using logistic regression models, without adjusting for cystatin C. Additionally, we used generalized additive models (GAM) to determine whether there was a dose-response relationship between glucose homeostasis markers and the risk of MCI (Hunsicker et al., 2016).

Step 4: Multiple linear regression model was constructed to evaluate the association between cystatin C and various glucose homeostasis indicators. The following covariates were examined as potential confounders: age, sex, smoking status, drinking status, hypertension status, diabetes status, and obesity status.

#### 2.5.3. Stratified and mediation analysis

To determine whether diabetes status affects the association between cystatin C and the risk of MCI, we conducted stratified analyses according to participants’ diabetes status (yes/no). To determine whether the glucose homeostasis indicators are potential mediators of the association between cystatin C and the risk of MCI, we performed a mediation analysis and estimated the total effect, direct effect, and indirect effect (Tingley et al., 2014). Direct effect indicates the effect of cystatin C on the risk of MCI after controlling for the glucose homeostasis indicator, and indirect effect is the estimated effect of cystatin C through the glucose homeostasis indicator. Mediation effect was calculated as the percentage of indirect effect (mediated by glucose homeostasis indicators) divided by total effect. The mediation analysis is performed using the *mediation* package of R, and its *mediate* function can automatically detect the type of models used for the mediator and outcome models and calculates the estimates of the average causal mediation effect (ACME) and other quantities of interest via the general algorithms described in Imai et al., 2010.

All statistical tests were two-sided, and *p*-values <0.05 were considered statistically significant. All analyses and visual outputs were performed using R software (Version 4.0.5) with packages “mice,” “mgcv,” “tidyverse,” “mediation,” and “ggplot2.”

## 3. Results

### 3.1. Demographic characteristics

**FIGURE 2:**
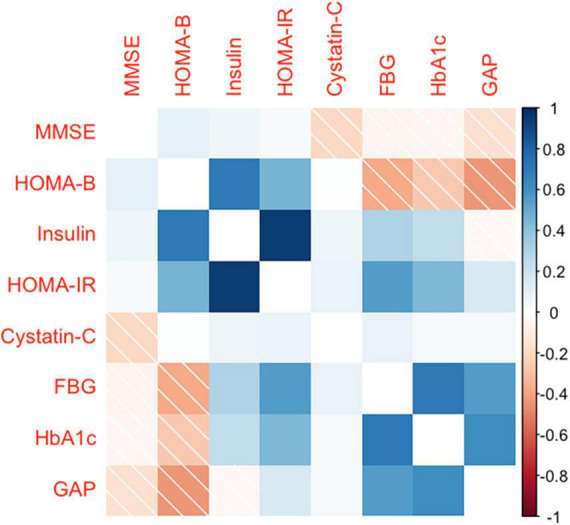
Spearman’s rank correlation coefficient between MMSE score, glucose homeostasis indicators, and cystatin C. MMSE, the Mini-Mental State Examination; FBG, fasting blood glucose; HbAlc, glycated hemoglobin; GAP, glycated albumin percentage (glycated albumin/albumin); HOMA-IR, homeostatic model assessment of insulin resistance; HOMA-β, homeostatic model assessment of beta cell function.

A total of 553 participants were enrolled in this study. A total of 514 participants were included in the analysis after excluding 39 participants who did not complete the questionnaire, did not retain blood samples, and aged <50 years (Table 1). The mean (SD) age and BMI were 64.3 (8.2) years and 26.4 (3.7) kg/m^2^, respectively; the mean (SD) MMSE score for all participants was 26.4 (4.1), with 19.4 (3.8) for the MCI group and 27.6 (2.7) for the cognitively normal group. In terms of education level, 70.6% of participants reported completing junior high school or a higher level of education. Most participants reported never having been a smoker (71.4%) or drinker (64.4%). Approximately 71.0% of the participants had an annual household income between 10 and 100 thousand Chinese Yuan (CNY). Of the participants, 57.6% had hypertension, 28.0% had diabetes, and 30.7% had obesity. Table 1 displays the median (25th and 75th percentiles) for glucose homeostasis indicators and cystatin C. Figure 2 displays the spearman coefficients of correlation between MMSE scores, cystatin C, and glucose homeostasis indicators. We noted a strong positive correlation of HOMA-IR and HOMA-β with insulin. Correlation of insulin with FBG is influenced by participants’ diabetic status (non-diabetes: *r* = 0.33, diabetes: *r* = 0.02).

**TABLE 1:** Demographics and participants’ health status (*n* = 514).

	Overall	Normal (*n* = 438)	MCI (*n* = 76)	*p*-Value
Age, years, mean ± SD	64.3 ± 8.2	63.5 ± 7.7	69.1 ± 9.5	<0.001
BMI, kg/m^2^, mean ± SD	26.4 ± 3.7	26.3 ± 3.7	26.5 ± 3.5	0.635
MMSE, mean ± SD	26.4 ± 4.1	27.6 ± 2.7	19.4 ± 3.8	<0.001
Sex, female (%)	324 (63.0)	278 (63.5)	46 (60.5)	0.717
Education, *n* (%)				0.009
Illiteracy	49 (9.5)	35 (8.0)	14 (18.4)	
Primary school	102 (19.8)	85 (19.4)	17 (22.4)	
Junior high school or above	363 (70.6)	318 (72.6)	45 (59.2)	
Household Income, 1,000 CNY, *n* (%)				0.043
<10	99 (19.3)	81 (18.5)	18 (23.7)	
10−100	365 (71.0)	309 (70.5)	56 (73.7)	
>100	50 (9.7)	48 (11.0)	2 (2.6)	
Smoking status, *n* (%)				0.702
Never	367 (71.4)	315 (71.9)	52 (68.4)	
Former	44 (8.6)	36 (8.2)	8 (10.5)	
Current	103 (20.0)	87 (19.9)	16 (21.1)	
Drinking status, *n* (%)				0.750
Never	331 (64.4)	280 (63.9)	51 (67.1)	
Former	18 (3.5)	15 (3.4)	3 (3.9)	
Current	165 (32.1)	143 (32.6)	22 (28.9)	
Hypertension, *n* (%)				0.210
No	218 (42.4)	191 (43.6)	27 (35.5)	
Yes	296 (57.6)	247 (56.4)	49 (64.5)	
Diabetes, *n* (%)				0.213
No	370 (72.0)	320 (73.1)	50 (65.8)	
Yes	144 (28.0)	118 (26.9)	26 (34.2)	
Obesity, *n* (%)				0.360
Normal	121 (23.5)	104 (23.7)	17 (22.4)	
Overweight	230 (44.7)	201 (45.9)	29 (38.2)	
Obesity	158 (30.7)	129 (29.5)	29 (38.2)	
Underweight	5 (1.0)	4 (0.9)	1 (1.3)	
FBG, mmol/L*	5.7 (5.3, 6.6)	5.60 (5.3, 6.5)	5.9 (5.4, 7.6)	0.071
Insulin, mU/L*	7.2 (4.8, 10.9)	7.20 (4.9, 10.8)	7.0 (4.7, 11.0)	0.904
HbA1c, %*	5.8 (5.5, 6.2)	5.80 (5.4, 6.2)	5.8 (5.5, 6.4)	0.147
GAP, %*	14.0 (13.2, 15.6)	13.9 (13.1, 15.4)	14.9 (13.7, 17.0)	0.002
HOMA-IR*	1.9 (1.3, 3.1)	1.94 (1.3, 3.0)	2.0 (1.4, 3.4)	0.547
HOMA-β*	62.5 (41.2, 91.6)	62.5 (42.1, 93.4)	61.1 (33.8, 78.7)	0.133
Cystatin C, mg/L*	0.9 (0.8, 1.0)	0.87 (0.8, 1.0)	1.0 (0.85, 1.2)	<0.001

*Median (Q1, Q3). MCI, mild cognitive impairment; MMSE, the Mini-Mental State Examination; BMI, body mass index; FBG, fasting blood glucose; HbAlc, glycated hemoglobin; GAP, glycated albumin percentage (glycated albumin/albumin); HOMA-IR, homeostatic model assessment of insulin resistance; HOMA-β, homeostatic model assessment of beta cell function; CNY, Chinese Yuan.

### 3.2. Effects of cystatin C on the risk of MCI

Table 2 summarizes the associations between cystatin C and the risk of MCI based on logistic regression models. Participants with cystatin C levels≥1.09 mg/L had a 1.98 (95% CI, 1.05, 3.69) times higher risk of MCI than those with cystatin C levels <1.09 mg/L after multivariable-adjusted. However, after additional adjustment for blood glucose-related indicators (e.g., FBG, GAP, HbA1c), this association was no longer significant. Interestingly, the effect of cystatin C in increasing the risk of MCI was found only in the diabetes subgroup, and the OR of cystatin C in the diabetes subgroup was higher compared to the overall population, with a OR of 3.27 (95% CI, 1.01, 10.84).

**TABLE 2:** Association between cystatin C and the risk of MCI based on logistic regression.

Additional adjusted covariates	ORs (95%CI) of cystatin C		
	**All participants (*n* = 514)**	**Non-diabetes (*n* = 370)**	**Diabetes (*n* = 144)**
None	**1.98 (1.05, 3.69)**	1.80 (0.77, 4.06)	**3.27 (1.01, 10.84)**
FBG	1.88 (0.99, 3.52)	1.82 (0.78, 4.10)	2.71 (0.79, 9.35)
GAP	1.84 (0.97, 3.47)	1.75 (0.74, 4.02)	2.92 (0.85, 10.17)
HbA1c	1.86 (0.98, 3.48)	1.77 (0.76, 4.01)	2.70 (0.79, 9.39)
HOMA-β	**2.14 (1.13, 4.03)**	1.90 (0.80, 4.40)	**3.98 (1.18, 14.22)**
HOMA-IR	**1.97 (1.04, 3.71)**	1.83 (0.78, 4.18)	3.05 (0.90, 10.60)
Insulin	**2.10 (1.10, 3.95)**	1.85 (0.78, 4.24)	**3.77 (1.12, 13.30)**

Values are presented as odd ratios (95% confidence interval) of cystatin C ≥ 1.09 mg/L. Cystatin C < 1.09 mg/L as reference. All the models adjusted for age, sex, smoking status, drinking status, education, household income, hypertension status, and obesity status. FBG, fasting blood glucose; HbAlc, glycated hemoglobin; GAP, glycated albumin percentage (glycated albumin/albumin); HOMA-IR, homeostatic model assessment of insulin resistance; HOMA-β, homeostatic model assessment of beta cell function. Bold values indicate the statistical significance.

### 3.3. Effects of glucose homeostasis on the risk of MCI

Table 3 summarizes the associations between glucose homeostasis indicators and the risk of MCI. Natural log transformation was performed for insulin, HOMA-IR, and HOMA-β to adjust the apparent skewed distribution. High levels of FBG, GAP, and HbA1c were significantly associated with increased risk of MCI, with ORs (95% CI) of 1.16 (1.02, 1.31), 1.11 (1.04, 1.19), and 1.31 (1.04, 1.63), respectively. High levels of HOMA-β were significantly associated with a decreased risk of MCI, with an OR (95% CI) of 0.59 (0.38, 0.89). Compared to the models adjusted for cystatin C, the ORs of the glucose homeostasis indicators were slightly increased in the models unadjusted for cystatin C. Similarly, in the stratified analysis, the association between glucose homeostasis indicators and the risk of MCI only existed in the diabetic population. Furthermore, we used a generalized additive model to explore the non-linear association between glucose homeostasis indicators and the risk of MCI, and the analysis showed no nonlinear dose-response relationship (Supplementary Figure 1).

**TABLE 3:** Association between various glucose homeostasis indicators and the risk of MCI based on logistic regression.

Glucose homeostasis indicators	All participants (*n* = 514)		Non-diabetes (*n* = 370)		Diabetes (*n* = 144)	
	**OR (95%CI) ^a^**	**OR (95%CI) ^b^**	**OR (95%CI) ^a^**	**OR (95%CI) ^b^**	**OR (95%CI) ^a^**	**OR (95%CI) ^b^**
FBG	**1.17 (1.03, 1.32)**	**1.16 (1.02, 1.31)**	1.17 (0.61, 2.23)	1.19 (0.62, 2.26)	**1.33 (1.10, 1.66)**	**1.31 (1.07, 1.64)**
GAP	**1.12 (1.04, 1.20)**	**1.11 (1.04, 1.19)**	1.28 (0.98, 1.68)	1.27 (0.97, 1.67)	**1.21 (1.07, 1.37)**	**1.20 (1.06, 1.36)**
HbA1c	**1.34 (1.07, 1.66)**	**1.31 (1.04, 1.63)**	0.77 (0.28, 2.08)	0.81 (0.29, 2.23)	**1.86 (1.26, 2.91)**	**1.82 (1.21, 2.88)**
HOMA-β	**0.61 (0.4, 0.93)**	**0.59 (0.38, 0.89)**	0.87 (0.44, 1.70)	0.79 (0.39, 1.57)	**0.43 (0.20, 0.88)**	**0.40 (0.18, 0.82)**
HOMA-IR	1.10 (0.73, 1.65)	1.02 (0.66, 1.55)	0.98 (0.53, 1.80)	0.92 (0.49, 1.71)	1.44 (0.66, 3.09)	1.18 (0.51, 2.63)
Insulin	0.87 (0.53, 1.42)	0.79 (0.47, 1.30)	0.95 (0.49, 1.82)	0.88 (0.44, 1.71)	0.84 (0.34, 1.95)	0.66 (0.24, 1.60)

^*a*^Adjusted for age, sex, education, household income, smoking status, drinking status, hypertension status, and obesity status.

^*b*^Additional adjusted for cystatin C based on ^a^.

FBG, fasting blood glucose; HbAlc, glycated hemoglobin; GAP, glycated albumin percentage (glycated albumin/albumin); HOMA-IR, homeostatic model assessment of insulin resistance; HOMA-β, homeostatic model assessment of beta cell function. Bold values indicate the statistical significance.

### 3.4. Effects of cystatin C on glucose homeostasis

Table 4 summarizes the associations between cystatin C and glucose homeostasis indicators based on multiple linear regression models. Compared with the participants with cystatin C levels <1.09 mg/L, the levels of insulin, HOMA-β, and HOMA-IR were significantly high in the participants with cystatin C level ≥1.09 mg/L, with adjusted β (95% CI) of 0.22 (0.09, 0.34), 0.20 (0.06, 0.34), and 0.23 (0.09, 0.36), respectively. However, we did not find a significant association between cystatin C and FBG, GAP, or HbA1c in either the diabetic or non-diabetic population.

**TABLE 4:** Association between cystatin C and glucose homeostasis indicators based on multiple linear regression.

Glucose homeostasis indicators (dependent variable)	ß of cystatin C (95%CI)^a^		
	**All participants (*n* = 514)**	**Non-diabetes (*n* = 370)**	**Diabetes (*n* = 144)**
FBG	0.15 (−0.18, 0.49)	0.01 (−0.14, 0.17)	0.72 (−0.32, 1.75)
GAP	0.32 (−0.30, 0.95)	0.05 (−0.32, 0.42)	1.11 (−0.76, 2.99)
HbA1c	0.09 (−0.10, 0.27)	−0.07 (−0.17, 0.03)	0.47 (−0.10, 1.03)
HOMA-β	**0.20 (0.06, 0.34)**	**0.22 (0.07, 0.37)**	0.18 (−0.15, 0.50)
HOMA-IR	**0.23 (0.09, 0.36)**	**0.21 (0.05, 0.38)**	**0.37 (0.11, 0.64)**
Insulin	**0.22 (0.09, 0.34)**	**0.21 (0.06, 0.37)**	**0.30 (0.05, 0.55)**

^*a*^Adjusted for age, sex, smoking status, drinking status, hypertension status, diabetes status, and obesity status. FBG, fasting blood glucose; HbAlc, glycated hemoglobin; GAP, glycated albumin percentage (glycated albumin/albumin); HOMA-IR, homeostatic model assessment of insulin resistance; HOMA-β, homeostatic model assessment of beta cell function. Bold values indicate the statistical significance.

### 3.5. Mediation analyses

Given the significant correlation between HOMA-β and both cystatin C and MCI risk observed in the previous analyses, we further examined the potential mediating effect of HOMA-β on the relationship between cystatin C and MCI risk. Mediation analysis showed a significant negative mediating effect of HOMA-β (proportion mediated: −16%) in the relationship between cystatin C and MCI risk (Figure 1).

## 4. Discussion

In the present study, we found a positive association between high levels of cystatin C and an increased risk of MCI, with glucose homeostasis indicator such as HOMA-β playing a negative mediating role in this relationship. Elevated levels of glucose homeostasis indicators such as FBG, GAP, and HbA1c were also found to increase the risk of MCI, but we did not observe a significant association between cystatin C and these indicators.

The findings of the present study that higher levels of cystatin C were associated with an increased risk of MCI in middle-aged and older adults, which is consistent with previous researches (Levy et al., 2001; Sastre et al., 2004; Chuo et al., 2007; Yaffe et al., 2008; Chen et al., 2021). Interestingly, a large body of previous research has demonstrated that cystatin C is a neuroprotective factor (Gauthier et al., 2011) and proposed various mechanisms such as induction of autophagy (Tizon et al., 2010), inhibition of oligomerization (Olafsson et al., 1996), and amyloid fibril formation (Olafsson et al., 1996). These findings are contrast to the results of the present study and other population-based studies (Levy et al., 2001; Sastre et al., 2004; Chuo et al., 2007; Yaffe et al., 2008; Chen et al., 2021). There is currently no rational explanation for this. However, our results highlight the potential role of glucose homeostasis in this relationship. Our results found that the association between cystatin C and MCI risk was observed only in the diabetic population. Moreover, this association lost its significance after adjusting for additional indicators of glucose homeostasis, such as FBG, GAP, and HbA1c, which suggest that elevated blood glucose may play a crucial role in the observed relationship. Substantial epidemiological evidence indicates that the abnormal glucose homeostasis is an independent risk factor for MCI (Biessels et al., 2006; Pivovarova et al., 2016; Luo et al., 2022). Abnormal glucose homeostasis impairs cognitive function through multiple pathways such as microvascular damage (Chait and Bornfeldt, 2009), impaired glucose metabolism (Leao et al., 2020), and increased beta-amyloid deposition (Taylor et al., 2017). Recently, a meta-analysis that included 122 literatures showed that diabetes conferred a 1.25–1.91-fold excess risk for cognitive disorders (cognitive impairment and dementia); In addition, even prediabetes and changes of abnormal glucose homeostasis biochemical indicators predicted increased incidence of cognitive impairment and dementia (Xue et al., 2019). Furthermore, the mediation analysis in this study revealed a negative mediating effect of HOMA-β in the association between cystatin C and the risk of MCI, suggesting that HOMA-β may attenuate the impact of cystatin C on cognitive function. However, this causal relationship requires further confirmation through additional experimental and longitudinal studies.

The association between glucose homeostasis and cognitive function is clear, however, the causal relationship between glucose homeostasis indicators and cystatin C remains unclear. Study has found that elevated levels of cystatin C may increase the risk of type 2 diabetes (Sahakyan et al., 2011). Some studies have also indicated that cystatin C may participate in insulin resistance and diabetes-related pathological processes through oxidative stress and inflammation (Servais et al., 2008; Surendar et al., 2010). Cystatin C may increase the risk of diabetes by participating in interrelated processes of inflammation (Hu et al., 2004). However, there are also studies demonstrating that oxidative stress can induce the synthesis of mRNA and protein of cystatin C, which is key pathogenetic component of the diabetes (Surendar et al., 2010). In this study, we did not find a significant relationship between cystatin C and FBG, GAP, or HbA1c. Instead, we observed a significant association between cystatin C and insulin, HOMA-IR, and HOMA-β. The result is consistent with the previous research, which suggests that cystatin C is associated with insulin resistance, obesity, and metabolic syndrome (Surendar et al., 2010). Further experimental evidence and support are necessary to establish the association between cystatin C and glycemic stability indicators.

This study has some limitations. Firstly, due to its cross-sectional design, only correlational analysis can be conducted, and causal inferences cannot be drawn. Secondly, the sample size of this study is relatively small, and future research should validate our findings in larger samples. Thirdly, this study only used the MMSE to assess participants’ cognitive function, which may only capture basic cognitive functions and may be influenced by education level. Finally, as this study only included Chinese individuals, more diverse populations are needed to verify our results.

## 5. Conclusion

Elevated levels of cystatin C are associated with an increased risk of MCI. The glucose homeostasis indicator, HOMA-β, plays a negative mediating role in the relationship between cystatin C and MCI risk. Our findings require further experimental research for validation, as well as verification in larger samples.

## Data availability statement

The original contributions presented in this study are included in the article/Supplementary material, further inquiries can be directed to the corresponding author.

## Ethics statement

The studies involving human participants were reviewed and approved by the Institutional Ethics Committees of the Institute of Basic Medical Sciences for Chinese Academy of Medical Sciences. The patients/participants provided their written informed consent to participate in this study.

## Author contributions

KL: conceptualization, methodology, data-analysis and interpretation, and writing—original draft. JX, MZ, and YM: data collection and validation. JW: data-analysis. JZ, QZ, YL, and MY: data collection. QX: funding acquisition, writing—review and editing, and supervision. All authors contributed to the article and approved the submitted version.
