# Supplementary material for: Serum cystatin C and mild cognitive impairment: The mediating role of glucose homeostasis
Source: Front Aging Neurosci. 2023 Mar 28;15:1102762. doi: 10.3389/fnagi.2023.1102762 (PMC10086181; doi:10.3389/fnagi.2023.1102762)
Supplement: Supplementary file 1 [file Table_1.DOCX]

Supplementary Material

# Supplementary Figures


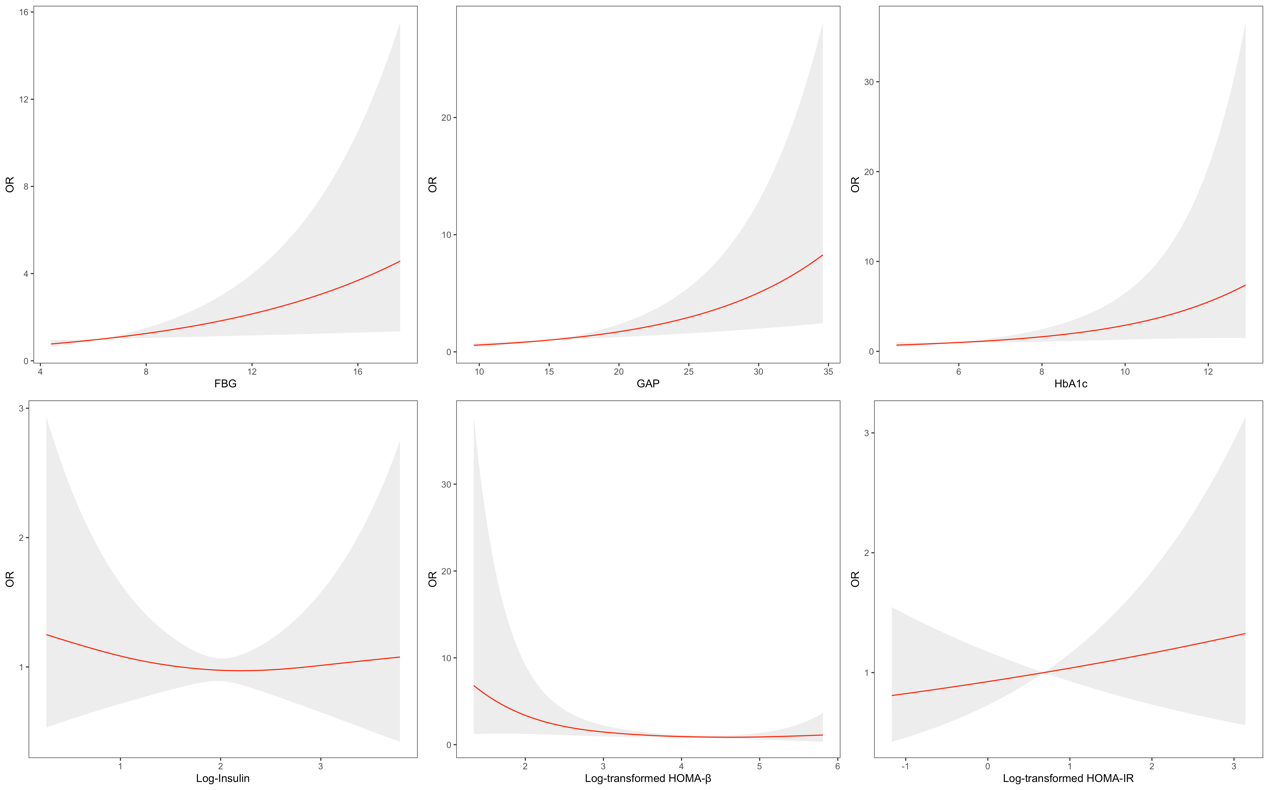


**Supplementary Figure 1.** Generalized additive models were used to model exposure-response relationships between glucose-homeostasis indicators and risk of MCI. The red line is the effect estimate, and shaded areas represent 95% CI. Abbreviations: FBG, fasting blood sugar; HbAlc, glycated hemoglobin; GAP, glycated albumin percentage (glycated albumin/albumin); HOMA-IR, Homeostatic Model Assessment of Insulin Resistance; HOMA-β, Homeostatic Model Assessment of Beta cell function.
